# Editorial: Language embodiment: Principles, processes, and theories for learning and teaching practices in typical and atypical readers

**DOI:** 10.3389/fpsyg.2023.1144176

**Published:** 2023-02-01

**Authors:** Laura M. Morett

**Affiliations:** Educational Studies in Psychology, Research Methodology, and Counseling, University of Alabama, Tuscaloosa, AL, United States

**Keywords:** language embodiment, gesture, handwriting, metaphor, event-related potential (ERP)

This Research Topic endorses the embodied view of language processing, which claims that, rather than being abstract, amodal, and arbitrary, the body plays a central role in shaping it (Glenberg and Kaschak, 2003). In particular, this Research Topic examines the implications of the embodied view for learning and teaching practices for typical and atypical populations, focusing in particular on its cognitive and neural mechanisms.

Several of the papers in this Research Topic focus on metaphor and closely related topics, an important theme in research on language embodiment (Gibbs, 2017; Hampe, 2017). Li et al. helped to disentangle the roles of simulation and abstraction in metaphor processing in Chinese by using event-related potentials (ERPs) to reveal that, in the verb processing stage, verb-object metaphors trigger simulation, whereas subject-verb metaphors trigger abstraction. This finding suggests that verbal metaphors are processed using both simulation and abstraction, and that metaphorical meaning is integrated in real-time during metaphor comprehension. Relatedly, Chen et al. investigated whether processing of metonymy, like metaphor, entails embodied simulation. Using eye-tracking, they found that readers take longer to arrive at a literal interpretation than a metonymic one when preceding information is weakly related to target words but not when it is strongly related to target words. Moreover, they found that preceding and spillover contextual information contributes to metonymy processing when spillover affects metonymy more than literal meaning. These findings provide insight into how sentential components contribute to metonymic processing of target words in Chinese, contributing to a model of metonymy processing.

Metaphor is pervasive in literary and scientific texts, so it is important to consider how it relates to embodied views of language processing within these contexts. Sun et al. used ERPs and event-related spectral perturbations (ERSPs) to investigate the processing of literary metaphors in modern Chinese poetry in comparison to non-literary conventional metaphors and literal expressions outside literary texts. They found more positive P200 and more negative N400 fronto-central components for literary metaphors than for other stimuli as well as increases in the delta and theta frequency bands during different time windows for literary metaphors. Together, these findings indicate that literary metaphor processing has distinct EEG spectral patterns and that it is characterized by early allocation of attention and conscious experience. Similarly, Tang et al. examined comprehension of scientific metaphors in first (L1; Chinese) and second (L2; English) language using ERPs. Relative to L1 Chinese scientific metaphors, L2 English scientific metaphors elicited more negative N400 and less positive late positive components (LPCs) in the parietal region as well as larger late negativities encompassing smaller areas of brain. These findings indicate that non-native and non-dominant language processing entails increased effort, decreased automaticity, and decreased sensitivity to the conventionality of metaphoric meanings. Finally, Huang et al. used hemifield presentation to investigate functional laterality during processing of scientific and conventional metaphors. The results support the fine-coarse coding hypothesis, which posits that the left hemisphere supports integration of the loosely associated domains of scientific metaphor. Moreover, compared to literal word pairs, conventional metaphors elicited higher LPCs during right visual field presentation, whereas scientific metaphors elicited lower LPCs during left visual field presentation. These findings suggest that processing mechanisms differ between novel and conventional metaphors and that right hemisphere plays a special role in novel metaphoric processing during the mapping stage.

Another important theme in this Research Topic is the relationship between domain- general and domain-specific processing. Zang et al. examined how L1–L2 language switching influences negation processing using a story reading and question verification paradigm in which the language was either kept the same or changed across tasks. They found that switching from L1 to L2 facilitated negative compared to affirmative responses, suggesting that domain-general mechanisms of inhibitory control are recruited simultaneously for L1–L2 language switching and negation. Seeking to reconcile conflicting results concerning the effect of multilingualism on executive function, Kim et al. examined the impact of task modality and found that it yields different patterns of performance between monolingual and multilingual participants, suggesting that distance of transfer from everyday language use may provide a viable explanation for these discrepancies. Yao R. et al. used a Stroop-like task to examine gesture-speech integration in deaf and hard of hearing adolescents. The results revealed that deaf and hard of hearing adolescents showed stronger effects of gesture-speech integration as well as semantic congruency than their hearing peers, revealing how sensory experience can influence language processing.

One method that has been instrumental in illuminating the relationship between domain- general and domain-specific processing in this Research Topic is priming, in which a (domain- general) prime influences processing of a (domain-specific) target. Zhou et al. examined the effects of musical primes with and without tempo changes on different classes of words using ERPs. When primed with tempo changes, state verbs and inanimate nouns elicited larger N400 amplitudes than action verbs and animate nouns, suggesting that such priming facilitates processing of action verbs and animate nouns due to the shared concept of motion across music and language. In a conceptually similar vein, Yao Z. et al. used picture-word semantic priming to examine the impact of social experience on processing of social abstract (e.g., friendship and betrayal) and emotional abstract (e.g., happiness and anger) words. Pictures of positive social scenes facilitated processing of positive social abstract words, and pictures of corresponding facial expressions and gestures facilitated processing of positive emotional abstract words, whereas no facilitatory priming was observed for negative pictures and social or emotional abstract words.

These findings provide evidence that social experience selectively affects processing of positive social and emotional language, providing a mechanism for the grounding of these concepts.

In addition to illuminating the mechanisms of embodied language processing, some papers in this Research Topic provide insight into how they can be leveraged to promote language comprehension in instructional settings. Li and Guan examined the effects of hand writing and drawing on development of orthographic representations. Behavioral and ERP data indicated that drawing facilitates visual word recognition in Chinese compared to viewing. Moreover, N170 amplitude correlated positively with N400 amplitude in the drawing condition, suggesting that hand movement may facilitate the neural correlates between early word recognition and later comprehension. Finally, Guan and Meng examined the effect of embodied morphological training by hand writing roots, dragging roots, and gesturing roots on children's L2 word learning. The results revealed that hand writing roots facilitated sound-meaning and word-sound form integration, whereas dragging and gesturing roots facilitated word form-meaning association, providing evidence that embodied morphological training contributes to children's L2 vocabulary acquisition.

In summary, the articles in this Research Topic reveal the inextricable relationship between language processing and embodied experience from several perspectives using cutting- edge behavioral and neuroscience methods. Importantly, they demonstrate how the embodied grounding of language can be leveraged to enhance learning in typically and atypically developing individuals, complementing other recent efforts in this regard (Boieblan, 2022; Hughes-Berheim et al., 2022; Morett et al., 2022). In doing so, they contribute to the growing literature providing insight into the myriad ways in which language processing is embodied and in which the mechanisms of its embodiment can be leveraged to enhance learning in individuals with diverse lived experiences.

## Author contributions

The author confirms being the sole contributor of this work and has approved it for publication.
